# Nonsystemic vasculitic neuropathy in a patient with IgG-monoclonal gammopathy of undetermined significance: A case report

**DOI:** 10.1097/MD.0000000000019036

**Published:** 2020-01-31

**Authors:** Ryuta Kinno, Yuyuko Osakabe, Seiya Takahashi, Shinji Kurokawa, Yoshiyuki Owan, Kenjiro Ono, Yasuhiko Baba

**Affiliations:** a Department of Neurology, Showa University Fujigaoka Hospital, Fujigaoka Aoba-ku, Yokohama-shi, Kanagawa; b Division of Neurology, Department of Medicine, Showa University School of Medicine, Hatanodai Shinagawa-ku, Tokyo.

**Keywords:** MGUS, nerve biopsy, nonsystemic vasculitic neuropathy, sensory ataxia

## Abstract

**Rationale:**

Monoclonal gammopathy of undetermined significance (MGUS) is a plasma cell proliferative disorder that consistently precedes multiple myeloma. Peripheral neuropathy in patients with IgG-MGUS tends to vary in clinical phenotype. We report a rare case of a patient with IgG-MGUS who had nonsystemic vasculitic neuropathy (NSVN).

**Patient concerns:**

A 56-year-old Japanese woman presented with progressive sensory ataxia with episodic paresthesia. Her clinical and laboratory values were compatible with IgG-MGUS. A nerve conduction study suggested possible chronic inflammatory demyelinating polyneuropathy. However, intravenous immunoglobulin therapy was not effective. A sural nerve biopsy specimen revealed mildly reduced myelinated fiber density and myelin ovoid formation, with epineural arterioles infiltrated by inflammatory cells.

**Diagnoses:**

We accordingly diagnosed her condition as NSVN.

**Interventions:**

She was accordingly started on oral prednisolone (40 mg/d) at 3 months after the onset of her neurological symptoms.

**Outcomes:**

At 1 year after the oral prednisolone treatment was begun, the patient's neurological symptoms showed no worsening.

**Lessons:**

These findings indicate NSVN as a possible cause of peripheral neuropathy in patients with IgG-MGUS. Cumulatively, our findings highlight the need for a nerve biopsy for peripheral neuropathy in patients with IgG-MGUS as a possible cause of NSVN. The early diagnosis of NSVN is expected to be beneficial for such patients.

## 1 Introduction

Monoclonal gammopathy of undetermined significance (MGUS) is a plasma cell proliferative disorder that consistently precedes multiple myeloma.^[1]^ It is characterized by a <10% plasma cell content in the bone marrow, a monoclonal (M) protein spike at ≤30 g/L, and no end-organ damage. Patients with MGUS are likely to experience peripheral neuropathy. Although the nature of the association between peripheral neuropathy and MGUS is not clear, it was reported that patients with IgM-related neuropathy often possess anti - myelin-associated glycoprotein (MAG) antibodies in the serum.^[2]^ In contrast, antibodies with this activity are usually absent in immunoglobin (Ig)G- and IgA-associated neuropathies, and these neuropathies tend to be more varied in their clinical phenotype.^[3]^ We report a rare case of a patient with IgG-MGUS who had nonsystemic vasculitic neuropathy (NSVN).

## 2 Case presentation

A 56-year-old Japanese female presented with painful paresthesia and numbness of her left thumb and 2nd and 3rd fingers. One month later, she experienced similar symptoms in her right 4th and 5th fingers. She noticed difficulty in walking with numbness in her left sole and clumsiness in her hands. These symptoms gradually worsened, and she presented at our department with painful paresthesia and numbness 8 months after the onset of symptoms.

On admission, the physical examination revealed that the patient was mentally alert with normal respiration and blood pressure. Her cranial nerve functions were intact, and no motor weakness was seen. Sensory nerve examinations demonstrated episodic paresthesia of both the palms and soles. Decreased position and vibration senses of both lower extremities were also recorded. The deep tendon reflex was decreased in the patient's left lower leg. She showed mild ataxia of the upper and lower extremities when her eyes were shut. The Romberg test result was positive. In summary, she had distal sensory disturbance and sensory ataxia. Indices of the extent of systemic infiltration, including the white blood cell count (3560/μL), erythrocyte sedimentation rate (20 mm/h), and C-reactive protein (<0.04 mg/dL) were normal.

Laboratory tests showed serum IgG-kappa monoclonal gammopathy without plasma cell expansion on bone marrow aspiration. The results of the following studies of the patient's serum samples were normal or negative: glucose level, antinuclear antibodies, rheumatoid factor, proteinase 3-antineutrophil cytoplasmic antibody, myeloperoxidase-specific antineutrophil cytoplasmic autoantibody, antibodies to SS-A and SS-B, angiotensin-converting enzyme, human immunodeficiency virus, antibody to varicella zoster, antineuronal antibodies, antiganglioside antibodies, and anti-MAG antibody. The protein content in the cerebrospinal fluid was 39 mg/dL with normal cellularity (3/μL; normal <10/μL), and oligoclonal IgG bands were absent.

Magnetic resonance imaging revealed no abnormalities in the patient's brain or spinal cord. Whole-body computed tomography scanning revealed no abnormalities suggestive of malignancy or lymph node involvement. Motor nerve conduction studies showed reduced distal amplitudes in the left tibial nerve, suggesting a conduction block (Table 1). We also observed slightly reduced conduction velocity and amplitude in the left ulnar and bilateral tibial nerves. Sensory nerve conduction studies demonstrated a reduced sensory nerve action potential (SNAP) in the right median and ulnar nerves. SNAPs were also not evoked in the left median, left ulnar, or left sural nerves. These electro-neurophysiologic observations for sensory nerves suggested an asymmetrical sensory-dominant polyneuropathy.

**Table 1:**
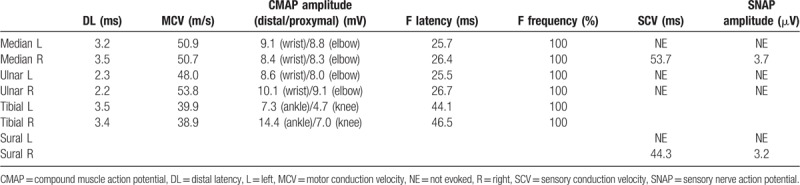
Results of nerve conduction study.

**Figure 1:**
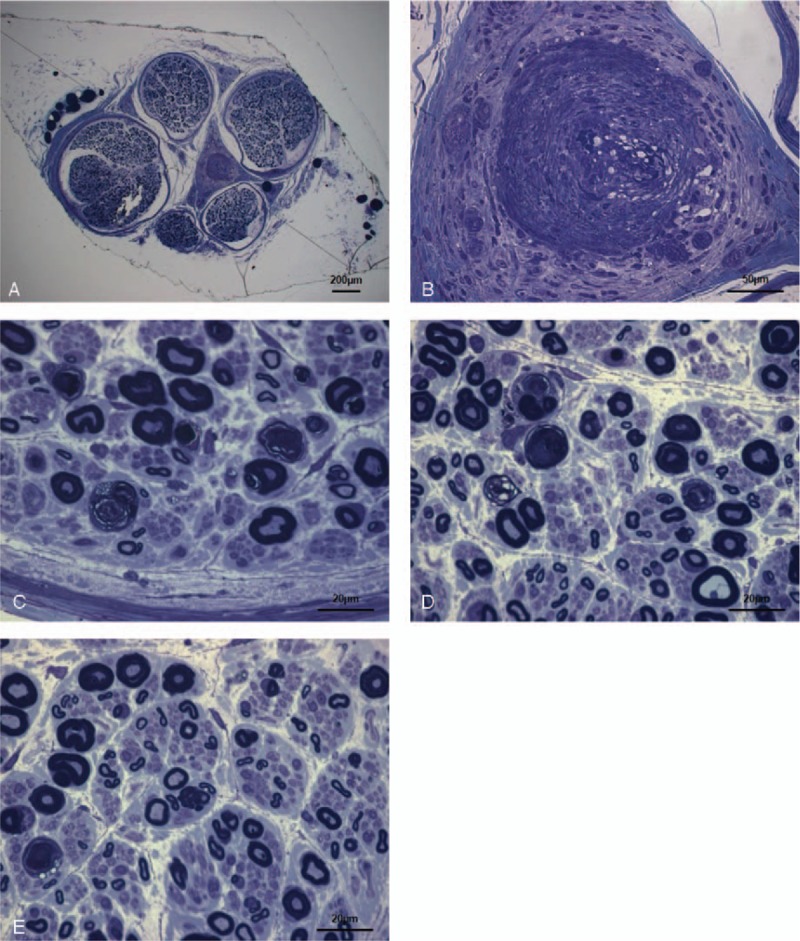
Sural nerve biopsy results, toluidine blue staining. Scale bars: A = 200 μm, B = 50 μm, C–E = 25 μm. Mild myelinated fiber loss and myelin ovoid formation with epineural arterioles infiltrated by inflammatory cells are seen.

We diagnosed a possible chronic inflammatory demyelinating polyneuropathy (CIDP) associated with MGUS and considered a treatment trial.^[4]^ We treated the patient with intravenous immunoglobulin (IVIg; 0.4 g/kg/d for 5 days). However, her neurological symptoms did not improve; they gradually worsened. For the evaluation of alternative causes of the patient's symptoms, we performed a sural nerve biopsy. Five fascicles with endoneurial edema were observed under toluidine blue staining (Fig. 1). This specimen revealed mildly reduced myelinated fiber density and myelin ovoid formation. Epineural arterioles infiltrated by inflammatory cells and accumulations of inflammatory cells were also observed. We found no abnormalities in Congo-red staining. These features were suggestive of vasculitis.

Considering the patient's clinical and pathological findings together, we diagnosed her condition as NSVN. She was accordingly started on oral prednisolone (40 mg/d) at 3 months after the onset of her neurological symptoms. At 1 year after the oral prednisolone treatment was begun, the patient's neurological symptoms showed no worsening.

## 3 Discussion

This is a rare case report of NSVN in a patient with IgG-MGUS. Our patient showed progressive sensory-ataxic polyneuropathy. Her laboratory examinations revealed serum IgG-kappa monoclonal gammopathy. Neither plasma cell expansion on bone marrow aspiration nor end-organ damage was observed, and this was compatible with the diagnosis of IgG-MGUS.^[1]^ Moreover, our patient's case satisfied the criteria for NSVN, including pathologically definite vasculitic neuropathy and no symptoms suggested underlying 'systemic’ vasculitis.^[5]^ Taken together, these findings suggest NSVN as a possible cause of peripheral neuropathy in patients with IgG-MGUS.

To the best of our knowledge, NSVN is a rare cause of peripheral neuropathy in patients with IgG-MGUS. When M proteins are detected in the setting of peripheral neuropathy, plasma cell dyscrasia with polyneuropathy, organomegaly, endocrinopathy, monoclonal protein and skin changes syndrome, AL amyloid neuropathy, multiple myeloma, and Waldenström macroglobulinemia must be considered in the differential diagnosis.^[2]^ These disorders must be excluded by bone marrow studies to determine the proportion of clonal cells and by imaging studies to eliminate osteolytic bone lesions, lymphadenopathy, and organomegaly.^[6]^ Indeed, all of these disorders were excluded in our patient's case by bone marrow and nerve studies. Because IgG-MGUS is not a common reason for peripheral neuropathy, alternative causes in such cases should be considered.^[2]^ We observed no other possible causes for our patient's peripheral neuropathy than NSVN, and we thus considered the NSVN as the probable cause of her peripheral neuropathy. Our experience suggests that a sural nerve biopsy should be performed when considering the diagnosis of NSVN as the possible cause of peripheral neuropathy in a patient with IgG-MGUS.

It is recommended that all patients with progressive NSVN and all those with definite, active vasculitis demonstrated in a recent nerve biopsy (irrespective of the clinical course) should be treated.^[5]^ Corticosteroid monotherapy is preferred for patients with NSVN who do not show ’rapid progression’ (ie, new motor or sensory deficits within 4 weeks of presentation). For patients with rapidly progressive NSVN and for NSVN patients who are steroid-refractory, combination therapy such as that using corticosteroids with cyclophosphamide, methotrexate, or azathioprine is advised. In contrast, no evidence for the treatment of IgG-MGUS-associated peripheral neuropathy has been described.^[2]^ Non-IgM-related peripheral neuropathy presenting with features similar to CIDP may be treated as CIDP with plasmapheresis, IVIg, and prednisone. Indeed, the effectiveness of IVIg therapy for such patients has been demonstrated.^[7,8]^ A causal relationship should generally not be considered in patients with non-IgM M proteins with peripheral neuropathy and features not resembling CIDP. In most of these patients, it is believed that the relationship between the M protein and neuropathy may be coincidental and that there is greater potential for harm with therapy. In our patient's case, IVIg therapy did not show any efficacy, but corticosteroid monotherapy improved her neurologic symptoms. Cumulatively, our findings recommend that a nerve biopsy be conducted as a test for peripheral neuropathy in patients with IgG-MGUS as a possible cause of NSVN. The early diagnosis of NSVN is expected to be beneficial for such patients.

## Acknowledgments

We thank Dr. Jun Shimizu and the staff of the Department of Neurology at the University of Tokyo for their excellent comments.

## Author contributions

**Conceptualization:** Ryuta Kinno, Yuyuko Osakabe, Yasuhiko Baba.

**Data curation:** Ryuta Kinno, Yuyuko Osakabe, Seiya Takahashi, Shinji Kurokawa, Yoshiyuki Owan, Yasuhiko Baba.

**Funding acquisition:** Ryuta Kinno.

**Supervision:** Kenjiro Ono, Yasuhiko Baba.

**Writing – original draft:** Ryuta Kinno, Yuyuko Osakabe.

**Writing – review & editing:** Ryuta Kinno, Yuyuko Osakabe, Seiya Takahashi, Shinji Kurokawa, Yoshiyuki Owan, Kenjiro Ono, Yasuhiko Baba.
